# Reparative effect of different dietary additives on soybean meal-induced intestinal injury in yellow drum (*Nibea albiflora*)

**DOI:** 10.3389/fimmu.2023.1296848

**Published:** 2023-12-08

**Authors:** Shipeng Ma, Ligai Wang, Yanqing Zeng, Peng Tan, Ruiyi Chen, Weihua Hu, Hanxiang Xu, Dongdong Xu

**Affiliations:** ^1^ Fisheries College, Zhejiang Ocean University, Zhoushan, China; ^2^ Key Laboratory of Mariculture and Enhancement, Zhejiang Marine Fisheries Research Institute, Zhoushan, China

**Keywords:** *Nibea albiflora*, soybean meal, dietary additive, intestinal injury, reparative effect

## Abstract

Soybean meal (SBM) is an acceptable replacement for unsustainable marine fish meal (FM) in aquaculture. However, we previously reported that high dietary SBM supplementation causes intestinal inflammatory injury in yellow drum (*Nibea albiflora*). Accordingly, a 4-week SBM-induced enteritis (SBMIE) in yellow drum trial was conducted first, followed by a 4-week additive-supplemented reparative experiment to evaluate the reparative effect of five additives on SBMIE in yellow drum. The control diet comprised 50% FM protein substituted with SBM. The additive-supplemented diet was added with 0.02% curcumin (SBMC), 0.05% berberine (SBM-BBR), 0.5% tea polyphenols (SBM-TPS), 1% taurine (SBM-TAU), or 0.8% glutamine (SBM-GLU) based on the control diet, respectively. The weight gain (WG), specific growth rate (SGR), feed efficiency ratio (FER), and survival rate (SR) of fish fed the additive-supplemented diets were significantly higher than those of fish fed the SBM diet. The WG, SGR, and FER of fish fed the SBMC, SBM-GLU and SBM-TAU diets were significantly higher than those of fish fed other diets. Moreover, fish fed the additive-supplemented diets SBMC and SBM-GLU, exhibited significantly increased intestinal villus height (IVH), intestinal muscular thickness (IMRT), and intestinal mucosal thickness (IMLT) and significantly decreased crypt depth (CD) in comparison with those fed the SBM diets. The relative expression of intestinal tight junction factors (*ocln, zo1*), cytoskeletal factors (*f-actin, arp2/3*), and anti-inflammatory cytokine*s (il10, tgfb)* mRNA was remarkably elevated in fish fed additive-supplemented diets than those of fish fed the SBM diet. Whereas, the relative expression of intestinal myosin light chain kinase (*mlck*) and pro-inflammatory cytokines (*il1, il6, tnfa*) mRNA was markedly lower in fish fed the additive-supplemented diets. The highest relative expression of intestinal *ocln*, *f-actin*, and *arp2/3* and the lowest relative expression of intestinal *mlck* were found in fish fed the SBMC diet. Hence, all five dietary additives effectively repaired the intestinal injury induced by SBM, with curcumin exhibiting the strongest repair effect for SBMIE in yellow drum.

## 1 Introduction

Soybean meal (SBM) is a good-quality plant protein source commonly used as a substitution to unsustainable fish meal (FM) in aquaculture feed because of its reasonable price, security of supply, and sustainability (1). However, the various anti-nutritional factors and unbalanced amino acid content in SBM in fish feed (2) could lead to a pro-inflammatory response in the distal intestine of carnivorous fish (3–7). Similarly, we previously reported that substituted of 45% FM protein with SBM protein reduced growth performance and induced intestinal inflammation in yellow drum (*Nibea albiflora*) (8). The primary associated manifestations include shortened intestinal mucosal folds, swollen of the lamina propria and subepithelial mucosa, and intense infiltration of inflammatory cells, which, collectively, can reduce the nutrient absorption capacity of the intestine and decrease growth performance (5, 9). Hence, it is particularly important to develop strategies to minimize intestinal injury caused by high proportions of SBM supplementation in marine fish.

Countermeasures, including enhancing processing technology and cultivating soybean protein-tolerant fish varieties, have been implemented for the widely use of SBM in cultivated fish varieties (10). Functional feed additives have also been served to alleviate SBM-induced enteropathy (SBMIE) in fish (11). These additives are nutritional/non-nutritional compounds provided in fish diets that enhance the physicochemical properties of diets or the performance of the target species (12, 13). Previous research suggests that a myriad of additives exhibit beneficiary properties such as anti-inflammatory, antioxidant, and immunomodulatory and may thus have the capacity to relieve SBMIE symptoms by strengthening the anti-inflammatory and immune responses of fish (14–16). Curcumin and berberine are both traditional herbal extracts, which can enhance intestinal villus development in Nile tilapia (*Oreochromis niloticus*) (17) and protect the intestinal barrier function of fish by regulating the intestinal microbiota (18). Meanwhile, tea polyphenols are plant extracts, which can attenuate the impairment of intestinal barrier function in fish by upregulating the expression of tight junction proteins and adhesion junction proteins (19). Taurine and glutamine are important functional amino acids, which has immune-enhancing and systemic nitrogen balance maintaining properties. Besides, taurine can improve intestinal homeostasis by regulating intestinal microbiota in mouse (20). Glutamine enhanced the intestinal tissue oxygenation and/or brush barrier function, as well as altered inflammatory processes in red drum (*Sciaenops ocellatus)* (21). Hence, all of these additives have great potential in the treatment of intestine injury in fish.

Yellow drum (*N. albiflora*) has become an important species for marine aquaculture in the East China Sea region due to its taste, rich nutrient content, and rapid growth (22). The successful artificial breeding of yellow drums has enabled their intensive cultivation, particularly in the coastal provinces of eastern China. We previously found that high dietary SBM supplementation causes intestinal inflammatory injury in yellow drum (8). Hence, in this study, we evaluated the effects of curcumin, berberine, tea polyphenols, glutamine, and taurine as potential feed additives on growth performance, whole-body nutritional composition, intestinal structure, and immune-related gene expression in yellow drum fed a high proportion of SBM diets. Collectively, this study can provide an effective solution for repairing intestinal injuries induced by SBM in marine fish.

## 2 Materials and methods

### 2.1 Experimental diets

In the current study, six isonitrogenous (49.95%) and isolipid (12.50%) diets were formulated using FM and SBM as the primary protein sources, fish oil and soybean oil as the primary lipid sources, and wheat flour as the carbohydrate source, according to the formulas described by Wang et al. (2016) (23). In the control diet, 50% of the FM protein was substituted with SBM protein (**Table 1**), whereas the other five treatment diets were supplemented with 0.02% curcumin (SBMC), 0.05% berberine (SBM-BBR), 0.5% tea polyphenols (SBM-TPS), 1% taurine (SBM-TAU), or 0.8% glutamine (SBM-TAU) based on the control diet.

**Table 1:** Ingredients and composition of the experimental diets (%, dry-matter basis).

Ingredients	Experimental diets					
	SBM ^1^	SBMC ^2^	SBM-BBR ^3^	SBM-TPS ^4^	SBM-GLU ^5^	SBM-TAU ^6^
Fish meal ^7^	25.00	25.00	25.00	25.00	25.00	25.00
Soybean meal	28.59	28.59	28.59	28.59	28.59	28.59
Soy protein concentrate	8.00	8.00	8.00	8.00	8.00	8.00
Wheat gluten meal	11.00	11.00	11.00	11.00	11.00	11.00
Wheat flour	4.75	4.75	4.75	4.75	4.75	4.75
Fish oil	4.75	4.75	4.75	4.75	4.75	4.75
Soybean oil	1.00	1.00	1.00	1.00	1.00	1.00
Soybean lecithin	2.00	2.00	2.00	2.00	2.00	2.00
Premix for marine fish ^8^	0.30	0.30	0.30	0.30	0.30	0.30
Choline chloride	0.50	0.50	0.50	0.50	0.50	0.50
Monocalcium phosphate	0.50	0.50	0.50	0.50	0.50	0.50
**Additive**	0.00	0.02	0.05	0.50	0.80	1.00
Feeding attractant ^9^	0.10	0.10	0.10	0.10	0.10	0.10
Mildew preventive ^10^	0.20	0.20	0.20	0.20	0.20	0.20
DL-Methionine	0.10	0.10	0.10	0.10	0.10	0.10
L-Lysine	1.50	1.50	1.50	1.50	1.50	1.50
Sodium carboxymethyl cellulose	3.72	3.70	3.66	3.21	2.91	2.71
Cellulose	96.28	96.30	96.34	96.79	97.09	97.29
Total	100.00	100.00	100.00	100.00	100.00	100.00
nutrient level (dry matter basis)						
Crude protein	49.95	50.22	49.93	49.93	50.34	50.56
Crude lipid	12.50	12.24	12.51	12.40	12.82	12.37
Crude ash	7.92	7.75	7.95	8.12	7.98	8.25

^1^SBM: As control diet, 50% of the fish meal protein was replaced by soybean meal.

^2-6^The additive-supplemented diets: Supplemented with 0.02% curcumin (SBMC), 0.05% berberine (SBM-BBR), 0.5% tea polyphenols (SBM-TPS), 1% taurine (SBM-TAU), or 0.8% glutamine (SBM-GLU) based on the control diet.

^7^Fish meal: Peruvian steamed fishmeal, Peru.

^8^Premix for marine fish: includes vitamin premix and mineral premix. vitamin premix (mg kg^-1^ diet); VB_1_, 25; VB_2_, 36.7; VA, 32; VE, 120; VD, 35; VK_3_, 5.1; VC, 142; VB_6_, 20; VB_12_, 0.1; VH, 1.2; VB_5_, 60; VB_9_, 20; VB_3_, 200; inositol, 792. mineral premix (mg kg^-1^ diet): magnesium sulfate, 1826; ferrous sulfate, 119; zinc sulfate, 76; manganese sulfate, 44; cobalt chloride, 2; potassium iodide, 0.8; copper sulfate, 1; sodium chloride, 100; monopotassium phosphate, 233.2; monosodium phosphate, 137.0.

^9^Feeding attractant: glycine:betaine = 1:3.

^10^Mildew preventive: fumaric acid:calcium propionate = 1:1.

### 2.2 Experimental fish and feeding management

Juvenile yellow drums obtained from the research station of the Marine Fisheries Institute of Zhejiang Province (Xixuan Island, Zhoushan, Zhejiang Province, China) were acclimated in an indoor flow-through aquaculture system for two weeks before initiating the feeding trial. A total of 450 yellow drum juveniles (initial weight: 6.65 ± 0.02 g) were transferred to 18 cylindrical fiberglass tanks (capacity: 1000 L) with 25 individuals per tank. Diets were randomly assigned to tanks in triplicates. The experiment was divided into two stages: week 1–4, when all fish were fed the SBM diet to induce intestinal inflammation; and week 4–8, when all fish, excluding the control group, were fed the additive-supplemented diets. All fish were fed twice daily at 7:00 and 14:00. The following parameters were maintained throughout the experimental period: temperature, 26–28 °C; salinity, 27.0 ± 1.0 g/L; unionized ammonia nitrogen< 0.05 mg/L; dissolved oxygen > 6.0 mg/L. The fish tanks were cleaned every two weeks, at which point the fish were removed and weighed to adjust the feeding amount.

### 2.3 Sample collection

After the feeding experiment, the fish were fasted for 24 h, the total number of fish in each tank was counted, and the survival rate (SR) was calculated. The weight of each tank fish was measured and then the weight gain rate (WG) and specific growth rate (SGR) of the fish were calculated. Fish were anesthetized with MS-222, and 12 individuals were randomly selected from each tank, and their total length, body length, and body weight were measured to calculate the condition factor (CF). Appropriately sized posterior intestinal tissue was cleaned with phosphate-buffered saline (PBS), and excess connective tissue was removed and fixed in 4% paraformaldehyde solution. Hindgut tissues were immersed in lyophilized vials containing RNA preservation solution (Solarbio, Beijing, China), flash-frozen in liquid nitrogen, and subsequently transferred to a −80°C refrigerator for storage. In addition, three fish were selected from each tank for the whole-body analysis.

### 2.4 Chemical analysis

The nutritional compositions of the experimental diets and whole fish were determined using the AOAC (1995) method. The samples were dried to a constant weight at 105 °C in a drying oven to measure the moisture content. Crude protein content was determined using a Kjeldahl nitrogen tester (BUCHI, KjeIFIex K-360, Switzerland). The crude fat content was determined using a Soxhlet extractor (FOSS Soxtec-2055, Sweden); the samples were heated in an electric furnace until they were smokeless and then cauterized to constant weight (4 h) using a Muffle furnace at 550°C to determine the ash content.

### 2.5 Histological analysis

The intestines and liver of three fish from each tank were collected for histometric evaluation. The fixed hindgut and liver samples were collected and dehydrated using a fully automated dehydrator. The dehydrated tissues were paraffin-embedded, and transversely cut into 5–6 μm tissue sections with a microtome and dried overnight.

The H&E (hematoxylin and eosin) staining process was divided into the following steps: dewaxing, staining, dehydration, transparency, and sealing. The sealed sections were imaged using a 250 FLASH digital pathology system (3DHISTECH, Hungary). Image-Pro Plus 6.0 software (Media Cybernetics) was used to analyze the intestinal villus height (IVH), intestinal muscle thickness (IMRT), intestinal mucosal thickness (IMLT), and crypt depth (CD) in each group (7, 24, 25).

### 2.6 RNA extraction and real‐time quantitative polymerase chain reaction

Total RNA was extracted and purified from distal intestinal tissue samples (approximately 50 mg) using the R1200 Total RNA Extraction Kit (Solarbio, Beijing, China) according to the manufacturer’s instructions. RNA concentration and purity were determined using an ND-2000 spectrophotometer (NanoDrop 2000; Wilmington, DE, USA). OD260/280 values between 1.8 and 2.0 indicated high-quality RNA samples, and the relative amounts of RNA and its integrity were detected by electrophoresis. The cDNA was synthesized with the PrimeScript RT reagent Kit with gDNA Eraser Kit (TaKaRa, Japan) and stored at −20°C for future use.

Primers were designed using Primer 3 (https://primer3.ut.ee/) based on the sequences of yellow drum-related genes in the yellow drum transcriptome (the target genes are shown in **Table 2**), and the primers were synthesized by the Zhejiang Shangya Biological Company. RT-qPCR was performed using TransStart Tip Green qPCR SuperMix (All Style Gold, Beijing, China) on a StepOnePlus real-time PCR system (Thermo Fisher Scientific, USA). The constitutively expressed ribosomal protein *actb*, which was shown to be stably expressed in the yellow drum, was selected as the housekeeping gene for sample normalization. After the values were normalized to *actb*, the fold change in transcript levels was determined by the relative quantitative method (2^−ΔΔCT^).

**Table 2:** Primers used for qRT-PCR.

Target gene	Forward primer (5′–3′)	Reverse primer (5′–3′)
*actb*	CCAACTCATTGGCATGGCTT	GATGCAACTGCAGAACCCTG
*f-actin*	AGGACAGCTACGTGGGAGAT	TGTTGGCTTTGGGGTTGAGT
*Arp2/3*	TACCCGGTGCTGTTTGTGTA	TGCACTGGTTTCTTCCTCCT
*il1*	TACTGTGCACCTGCCAAGAC	CTCTGTGCCCTTGTCCACTT
*il6*	TGAAGGCTCCGACGAAATG	GTCCAGTAGGCTAAACTGCTATC
*tnfa*	TGAAGAAGATGGTGCCCTTAC	GCCTGGAATCGAGCTCTAAAT
*il10*	CACTTTGTGGGCTACATCCA	GTTGAGGTATGCTGTGGTAGTC
*tgfb*	CGTCGCAGAACGCATCTATAA	CACGGCTATGATGTCCTGTATT
*ocln*	CCAGGCTACCAGGTGAAGAA	CTTCGGACAGGCGTGAAATC
*zo1*	TCACTCACCATGTTCCTCCC	CAGAAACACAGTTGGCTCCC
*mlck*	AGATGTGGAGGTGGTGGAAG	CGTGTATTTGGCATCGTCGT

actb, β-actin internal reference gene; f-actin, filamentous actin; arp2/3, arp2/3 complex; il1, interleukin 1; il6, interleukin 6; tnfa, tumor necrosis factor alpha; il10, interleukin 10; tgfb, transforming growth factor β; ocln, occludin; zo1, tight junction protein; mlck, myosin light chain kinase.

### 2.7 Calculations and statistical analysis

The following variables were calculated:

Survival rate (SR, %).


$$SR=N_{t}\times 100/N_{0}$$

Weight gain (WG, %).


$$WG=100\times \left(W_{t}-W_{0}\right)/W_{0}$$

Specific growth rate (SGR per day, %/d).


$$SGR=100\times \left(Ln\;W_{t}-Ln\;W_{0}\right)/t$$

Feed efficiency ratio (FER, %).


FER=100×weight gain/total amount of food consumed

Hepatosomatic index HSI (%).


$$HSI=100\times \left(liver\;weight/W_{t}\right)$$

Viscerosomatic index (VSI, %).


$$VSI=100\times \left(visceral\;weight/W_{t}\right)$$

Condition factor CF (g/cm^3^).


$$CF=100\times \left(body\;weight,\;g\right)/{\left(body\;lenght,cm\right)}^{3}$$

where W_t_ and W_0_ represent the final and initial fish weights, respectively; N_t_ and N_0_ are the final and initial numbers of fish in each tank, respectively; and t represents the trial period in days.

Data were analyzed by one-way analysis of variance (ANOVA) using SPSS23.0 software, Duncan’s multiple comparisons were performed when the data were normally distributed within groups, and a *P*< 0.05 was considered statistically significant. The experimental results were expressed as mean ± standard error (mean ± SEM).

## 3 Results

### 3.1 Growth, survival, and morphological indices

The fish fed additive-supplemented diets had remarkably elevated WG, SGR, SR, and FER than those of fish fed the SBM diet. The WG and SGR of fish fed SBM-TPS or SBM-BBR were remarkably lower than those of fish fed SBMC, SBM-GlU, or SBM-TAU(*P*< 0.05; **Table 3**).

**Table 3:** Analysis of growth performance, survival rate and morphological indicators.

Parameter	Group					
	SBM	SBMC	SBM-BBR	SBM-TPS	SBM-GLU	SBM-TAU
IBW (g)	6.67 ± 0.02	6.66 ± 0.02	6.65 ± 0.02	6.65 ± 0.01	6.66 ± 0.02	6.65 ± 0.02
MBW (g)	24.51 ± 0.34	25.54 ± 0.75	25.40 ± 0.68	25.06 ± 0.38	25.47 ± 0.59	25.44 ± 1.04
FBW (g)	53.37 ± 1.42^a^	61.16 ± 1.41^c^	57.18 ± 0.72^b^	56.44 ± 0.98^b^	60.29 ± 0.53^c^	60.28 ± 2.60^c^
WG (%)	700.65 ± 20.52^a^	817.74 ± 20.18^c^	759.51 ± 8.63^b^	748.24 ± 14.64^b^	805.46 ± 5.45^c^	806.12 ± 40.68^c^
SGR (%/d)	4.16 ± 0.05^a^	4.43 ± 0.04^c^	4.30 ± 0.02^b^	4.28 ± 0.03^b^	4.41 ± 0.01^c^	4.41 ± 0.09^c^
SR (%)	90.67 ± 2.31^a^	97.33 ± 2.31^b^	98.67 ± 2.31^b^	100.00 ± 0.00^b^	98.67 ± 2.31^b^	100.00 ± 0.00^b^
FER(%)	100.57 ± 10.01^a^	119.27 ± 2.04^b^	114.93 ± 2.99^b^	115.15 ± 1.15^b^	118.66 ± 2.46^b^	114.58 ± 4.61^b^
HSI (%)	1.65 ± 0.15^c^	1.64 ± 0.27^c^	1.54 ± 0.28^b^	1.41 ± 0.26^a^	1.62 ± 0.23^c^	1.39 ± 0.25^a^
VSI (%)	5.11 ± 0.32^b^	5.00 ± 0.62^b^	5.05 ± 0.29^b^	5.04 ± 0.69^b^	5.40 ± 0.52^c^	4.74 ± 0.51^a^
CF (g/cm^3^)	1.89 ± 0.11	1.97 ± 0.13	1.92 ± 0.10	1.91 ± 0.11	1.92 ± 0.14	1.94 ± 0.13

Data represent mean ± SEM (n = 3). Based on one-way ANOVA, values in the same row with different superscript letters are significantly different (P< 0.05). IBW, initial body weight; MBW (28d): metaphase body weight; FBW, final body weight; WG, weight gain rate; SGR, specific growth rate; SR, survival rate; FER, feed efficiency ratio; HIS, hepatosomatic index; VSI, viscerosomatic index; CF, condition factor.

The HSI and VSI differed significantly among the diets. The HSI of fish fed the SBM-BBR, SBM-TPS, and SBM-TAU diets were remarkably lower than that of fish fed the SBM diet (*P*< 0.05), whereas the HSI of fish fed the SBMC and SBM-GLU diets did not differ significantly from that of fish fed the SBM diet (*P* > 0.05). The VSI was significantly higher in fish fed the SBM-GLU diet than in those fed the SBM diet, and fish fed the SBM-TAU diet had significantly lower VSI than those fed the SBM diet.There were no significant differences between fish fed SBMC, SBM-BBR, or SBM-TPS diets and those fed SBM diets. The CF was not significantly different among the groups. (*P*< 0.05; **Table 3**).

### 3.2 Fish body composition

Excluding the fish in the SBM-GLU group, the fish fed the additive-supplemented diets had a remarkably lower moisture content than that of fish fed the SBM diet. The crude protein and lipid contents of fish fed the additive-supplemented diets, excluding the SBMC diet, were remarkably higher than those fed the control diet (*P*< 0.05; **Table 4**), whereas the crude lipid content of fish fed the SBMC diet did not differ significantly from that of fish fed the SBM diet (*P* > 0.05). Moreover, there was no significant difference in crude ash content between fish fed the additive-supplemented diets and those fed the SBM diet (*P* > 0.05).

**Table 4:** Whole fish body composition analysis.

Parameter	Group					
	SBM	SBMC	SBM-BBR	SBM-TPS	SBM-GLU	SBM-TAU
Moisture (%)	73.83 ± 0.27^b^	73.24 ± 0.38^a^	73.02 ± 0.94^a^	72.88 ± 0.13^a^	73.64 ± 0.81^ab^	73.06 ± 0.73^a^
Crude protein (%)	15.15 ± 0.20^a^	16.02 ± 0.30^bc^	16.51 ± 0.36^c^	16.00 ± 1.01^bc^	16.64 ± 0.19^c^	15.77 ± 0.26^b^
Crude lipid (%)	6.07 ± 0.38^a^	5.95 ± 0.13^a^	6.39 ± 2.00^b^	6.33 ± 1.23^b^	6.37 ± 0.37^b^	6.67 ± 1.67^b^
Ash (%)	3.43 ± 0.17	3.58 ± 0.14	3.54 ± 0.17	3.65 ± 0.21	3.43 ± 0.10	3.60 ± 0.27

Data represent mean ± SEM (n = 3). Based on one-way ANOVA, values in the same row with different superscript letters are significantly different (P< 0.05).

### 3.3 Muscle amino acid composition analysis

The fish fed the additive-supplemented diets (except for the SBMC diet) had remarkably increased essential amino acid (EAA), non-essential amino acid (NEAA), and total amino acid (TAA) contents than in those fed the SBM diet (*P*< 0.05; **Table 5**). The TAA contents of fish fed the SBM-GLU and SBM-BBR diets were the highest.

**Table 5:** Muscle amino acid composition analysis.

Parameter	Group					
	SBM	SBMC	SBM-BBR	SBM-TPS	SBM-GLU	SBM-TAU
Threonine (%)	3.49 ± 0.69^ab^	3.56 ± 0.17^abc^	4.12 ± 0.10^bc^	3.96 ± 0.09^bc^	4.69 ± 0.12^c^	3.67 ± 0.19^abc^
Valine (%)	3.72 ± 0.73^ab^	3.79 ± 0.21^ab^	4.37 ± 0.13^b^	4.17 ± 0.10^b^	4.40 ± 0.14^b^	3.90 ± 0.22^ab^
Methionine (%)	2.28 ± 0.26^ab^	2.52 ± 0.17^b^	2.89 ± 0.18^c^	2.84 ± 0.14^c^	3.00 ± 0.11^c^	2.54 ± 0.09^b^
Isoleucine (%)	3.49 ± 0.66^ab^	3.56 ± 0.21^abc^	4.11 ± 0.12^bc^	3.92 ± 0.12^bc^	4.15 ± 0.14^c^	3.66 ± 0.20^abc^
Leucine (%)	6.12 ± 1.19^ab^	6.23 ± 0.33^abc^	7.21 ± 0.26^bc^	6.94 ± 0.16^bc^	7.28 ± 0.24^c^	6.47 ± 0.36^abc^
Phenylalanine (%)	3.44 ± 0.66^ab^	3.52 ± 0.19^ab^	4.00 ± 0.18^b^	3.89 ± 0.10^b^	4.04 ± 0.13^b^	3.62 ± 0.17^ab^
Histidine (%)	1.57 ± 0.34^ab^	1.60 ± 0.07^abc^	1.89 ± 0.02c	1.79 ± 0.03bc	1.91 ± 0.08c	1.69 ± 0.11abc
Lysine (%)	7.26 ± 1.38^ab^	7.41 ± 0.41^abc^	8.50 ± 0.29^bc^	8.24 ± 0.18^bc^	8.63 ± 0.31^c^	7.68 ± 0.41^abc^
Arginine (%)	4.70 ± 0.92^ab^	4.78 ± 0.20^abc^	5.48 ± 0.15^bc^	5.33 ± 0.11b^c^	5.60 ± 0.13^c^	4.97 ± 0.25^abc^
EAA (%)	36.07 ± 6.81^a^	36.97 ± 1.92^a^	42.58 ± 1.31^c^	41.08 ± 1.01^c^	43.17 ± 1.39^c^	38.19 ± 1.99^b^
Serine (%)	3.22 ± 0.63^ab^	3.31 ± 0.16^abc^	3.87 ± 0.15^cd^	3.75 ± 0.10^bcd^	3.98 ± 0.11^d^	3.46 ± 0.18^abcd^
Glutamic acid (%)	12.61 ± 2.40^ab^	12.78 ± 0.64^ab^	14.89 ± 0.52^bc^	14.46 ± 0.38^bc^	15.29 ± 0.46^c^	13.44 ± 0.72^abc^
Glycine (%)	4.34 ± 0.88^ab^	4.50 ± 0.09^abc^	4.93 ± 0.29^bc^	5.05 ± 0.03^bc^	5.24 ± 0.18^c^	4.03 ± 0.06^a^
Alanine (%)	4.78 ± 0.90^ab^	4.89 ± 0.22^abc^	5.57 ± 0.22^bc^	5.48 ± 0.10^bc^	5.68 ± 0.16^c^	5.05 ± 0.20^abc^
Tyrosine (%)	2.75 ± 0.53^ab^	2.79 ± 0.16^ab^	3.21 ± 0.10^b^	3.08 ± 0.07^b^	3.25 ± 0.11^b^	2.89 ± 0.17^ab^
Asparagine (%)	8.13 ± 1.54^ab^	8.27 ± 0.45^abc^	9.53 ± 0.36^bc^	9.25 ± 0.23^bc^	9.69 ± 0.34^c^	8.57 ± 0.45^abc^
NEAA (%)	35.83 ± 6.86^a^	36.54 ± 1.63^a^	42.01 ± 1.64^b^	41.08 ± 0.89^b^	43.14 ± 1.17^c^	37.42 ± 1.75^ab^
TAA (%)	71.90 ± 13.67^a^	73.51 ± 3.52^a^	84.59 ± 2.95^c^	82.16 ± 1.90^b^	86.31 ± 2.55^c^	75.61 ± 3.74^ab^

Data represent mean ± SEM (n = 3). Based on one-way ANOVA, values in the same row with different superscript letters are significantly different (P< 0.05). EAA, essential amino acid; NEAA, non-essential amino acid; TAA, total amino acid.

### 3.4 Distal intestinal and liver micromorphology

**Figure 1:**
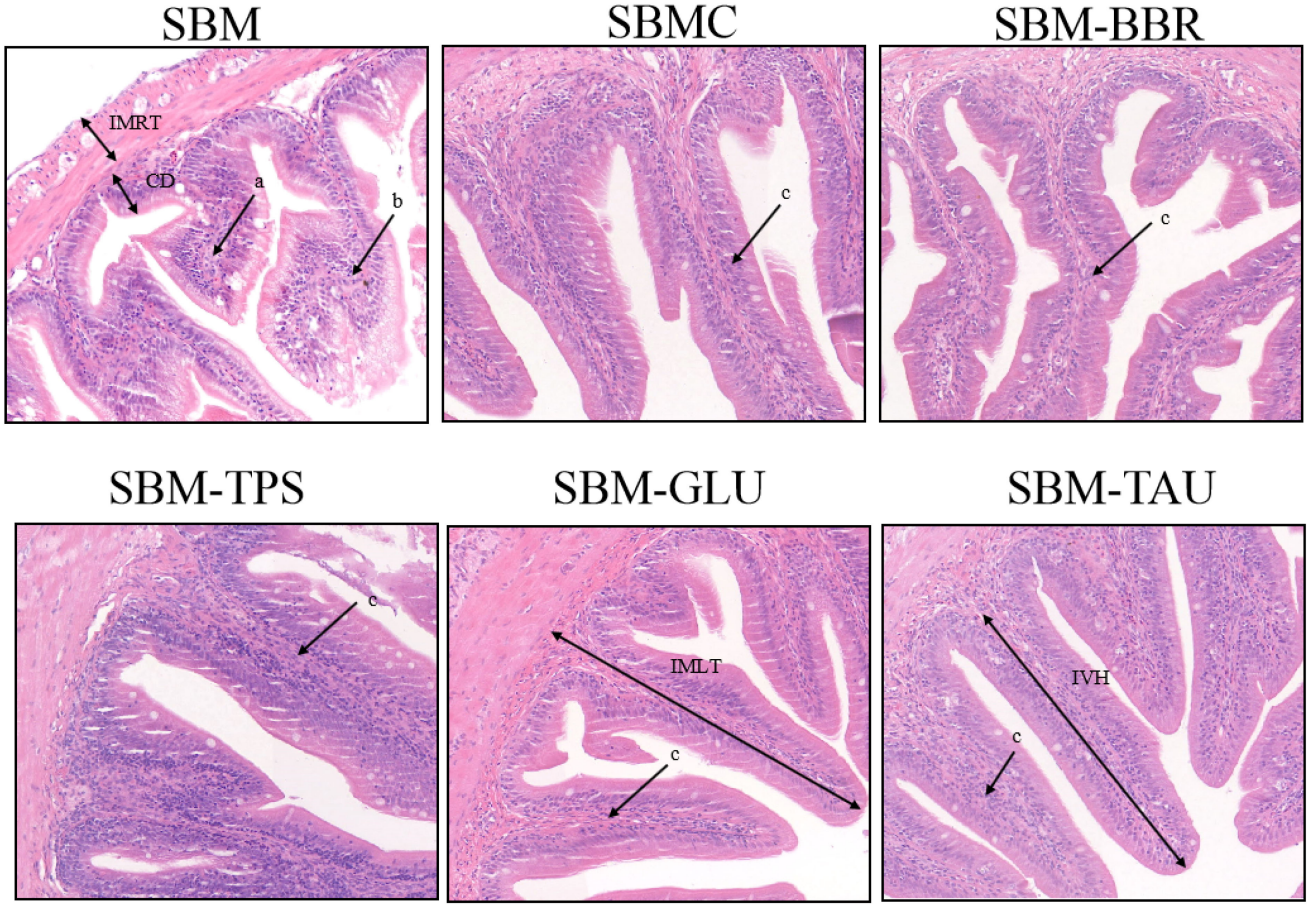
Representative histomorphological images from hematoxylin and eosin (H&E)-stained sections of the distal intestine. Scale Bar: 50 μm; (a) Widening of the lamina propria of the intestinal villi; (b) Intestinal villi atrophied, shortened; (c) intestinal villi are elongated and the lamina propria is thin; IVH, Intestinal villus height; IMRT, Intestinal muscular thickness; IMLT, Intestinal mucosal thickness; CD, Crypt depth.

Yellow drums fed the SBM diet exhibited intestinal fold atrophy and shortening, breakage, and detachment, enlargement of the intrinsic layer in the intestinal folds, and deepen the crypt depth, typical of SBMIE in the end of 8-week feeding trial. However, the intestinal morphology of the yellow drum significantly improved after consumption of the additive-supplemented diet (**Figure 1**). IVH, IMRT, and IMLT were significantly increased, and CD was remarkably decreased in fish fed an additive-supplemented diet, particularly the SBMC or SBM-GLU diets (*P*< 0.05; **Table 6**).

**Table 6:** Distal intestine tissue variable scores of the yellow drum fed the experimental diets.

Parameter	Group					
	SBM	SBMC	SBM-BBR	SBM-TPS	SBM-GLU	SBM-TAU
IVH (μm)	343.48 ± 6.94^a^	449.22 ± 3.40^c^	349.10 ± 13.31^a^	436.2 ± 7.70a^b^	455.03 ± 10.66^c^	405.37 ± 16.45^a^
IMLT(μm)	389.74 ± 3.05^a^	583.91 ± 19.40^c^	415.79 ± 22.04^ab^	540.65 ± 16.82^c^	532.57 ± 20.26^c^	465.81 ± 16.22^b^
IMRT(μm)	45.77 ± 2.54^a^	108.53 ± 7.15^d^	78.13 ± 1.52^c^	74.73 ± 3.88^c^	73.59 ± 2.01^c^	59.62 ± 2.18^b^
CD (μm)	77.84 ± 6.35^b^	46.14 ± 3.82^a^	46.31 ± 4.85^a^	50.78 ± 4.77^a^	48.78 ± 1.34^a^	48.67 ± 2.66^a^

Data represent mean ± SEM (n = 3). Based on one-way ANOVA, values in the same row with different superscript letters are significantly different (P< 0.05). IVH, intestinal villus height (μm); IMLT, Intestinal mucosal thickness; IMRT, Intestinal muscular thickness; CD, crypt depth (μm).

**Figure 2:**
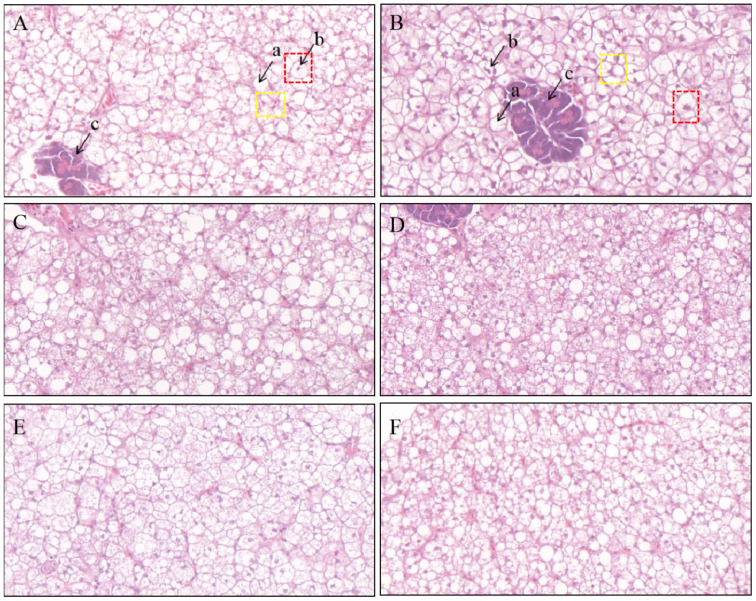
Representative histomorphological images from hematoxylin and eosin (H&E) stained sections of the liver. Scale Bar: 20 μm; **(A)** SBM; **(B)** SBMC; **(C)** SBM-BBR; **(D)** SBM-TPS; **(E)** SBM-GLU; **(F)** SBM-TAU; a: vacuolation; b: nucleus; c: portal vein; Red box: Normal liver cells; Yellow box: Liver cells with shifted nuclei.

A proportion of the hepatocytes from fish fed the SBM diet exhibited irregular geometry, severe vacuolization, intracellular localized nuclei, and solidification, whereas others lacked nuclei and exhibited broken cellular structures (**Figure 2A**). In contrast, fish fed the additive-supplemented diets exhibited markedly improved liver morphology, as evidenced by reduced cellular vacuolization, with most nuclei being centrally located and with intact cellular structures (**Figures 2B–F**). The repair effect was the most significant in the SBMC group.

### 3.5 Distal intestinal physical barrier-related gene expression

**Figure 3:**
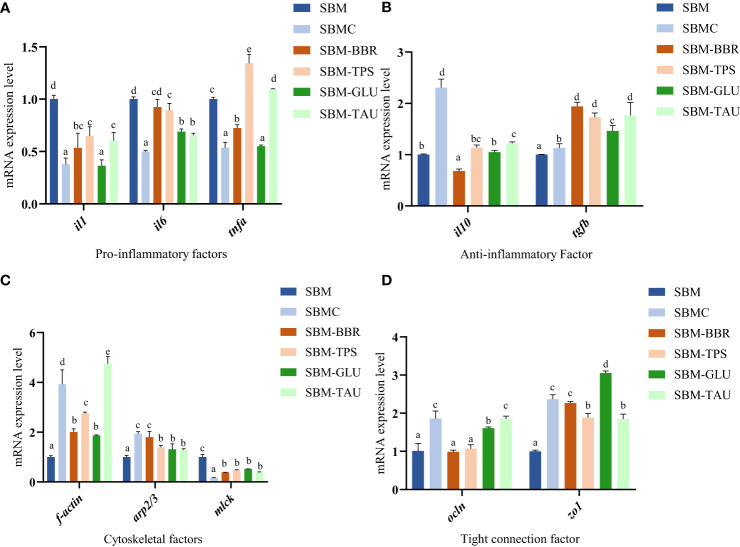
Relative mRNA expression of intestinal mucosal immunological barrier-related proteins in the distal intestine. Data represent as mean ± SEM (n = 3). According to the one-way ANOVA, values in the same row with different superscripts were significantly different (P< 0.05). SEM: standard error of the mean. il1, interleukin 1; il6, interleukin 6; tnfa, tumor necrosis factor alpha; il10, interleukin 10; tgfb, transforming growth factor β; f-actin, filamentous actin; arp2/3, arp2/3 complex; mlck, myosin light chain kinase; ocln, occludin; zo1, tight junction protein.

Overall, the expression of *il1*, *il6*, and *tnfa* mRNAs was significantly downregulated, whereas that of *il10* and *tgfb* was significantly upregulated in the distal intestines of fish fed the additive-supplemented diets than that of fish fed the SBM diet (*P*< 0.05; **Figures 3A, B**). Whereas, no significant difference was observed in *il6* expression or a decrease in *il10* mRNA expression in fish fed SBM-BBR. Moreover, there was an elevated expression levels of *tnfa* mRNA but no significant different expression level of *il10* mRNA in the SBM-TPS diet than those of fish fed the SBM diet (**Figures 3A, B**).

The mRNA expression levels of filamentous actin (*f-actin*), arp2/3 complex (*arp2/3*), and occludin (*ocln*) were remarkably higher in fish fed the additive-supplemented diets (*P*< 0.05; **Figure 3C**) than in the SBM-fed group. In particular, the increase in *ocln* expression was more significant in fish fed SBMC, SBM-GLU, and SBM-TAU diets. Moreover, the zonula occludens-1 (*zo1*) mRNA expression was significantly higher, whereas that of myosin light-chain kinase (*mlck*) was remarkably lower in fish fed the additive-supplemented diets than that of fish fed the SBM diet (*P*< 0.05; **Figure 3D**).

## 4 Discussion

In the present study, we evaluated the effectiveness of five feed additives on the repair of SBMIE in yellow drums by assessing the growth performance, intestinal histological morphology, and mRNA expression levels of intestinal barrier-related factors. High levels of SBM in feed can induce intestinal inflammation and injury in carnivorous fish, reducing the ability of the intestinal epithelium to uptake nutrients, and ultimately decreasing fish growth performance (2). However, the growth and survival of fish fed a high proportion of soybean meal diet can be significantly improved through the supplementation of functional feed additives (26, 27). In this study, the growth performance of fish fed diets provided with various additives were remarkably improved compared to those fed the SBM diet. The WG, SGR, and FER of the yellow drum fed SBMC, SBM-GlU, and SBM-TAU diets were the highest. This may be due to the additives reducing intestinal inflammation and increasing intestinal villus length and absorption area. Long villus are related to fine intestinal fitness, nutrient beneficiation, and uptake efficiency, thus improving growth performance (28, 29). This is further corroborated by the histomorphometric measurements of the intestinal tract from this study.

Replacing a significant proportion of FM protein with plant proteins can affect fish intestinal health and reduce crude protein and lipid contents, effectively diminishing the nutritional value of fish (30, 31). However, previous research has indicated that additives supplemented in high plant protein replacing fish meal diet can significantly improve fish body composition (21, 32). In the present study, the whole-body crude protein and crude lipid contents of fish fed the additive-supplemented diets were remarkably higher, whereas the moisture content was significantly lower than that of fish fed the SBM diet. This is likely due to additives that enhance the conversion of ingested food into whole-body proteins and lipids by improving intestinal morphology.

Assessing the changes in intestinal morphology is the most common and direct method for evaluating intestinal barrier function (33). Dietary SBM-induced pro-inflammatory responses are caused by injury to the physical barrier of the intestinal mucosa, subsequently exposing the otherwise protected layers of the mucosa to luminal ingredients, including pathogenic bacteria or other food antigens, thereby exacerbating the enteritis (34). Moreover, the intestine is intricately connected to the liver in terms of immunity and digestion; hence, intestinal injury can lead to liver injury, following the intestine’s exposure to harmful bacteria and metabolites (35–37). Nonetheless, the physical barrier of the intestinal mucosa can be improved by the ingestion of dietary additives that serve to repair intestinal injury (38, 39). As evidenced by the intestinal morphological indicators, the additives selected for this study significantly improved the intestinal integrity of yellow drums, which was characterized by increases in IVH, IMRT, IMLT, and CD. Similar results have been observed in *Epinephelus lanceolatus* (40) and *Oreochromis niloticus* (14), corroborating that additive-supplemented diets elicit reparative effects on the intestine injury of the yellow drum. Additionally, the degree of liver injury in fish was markedly ameliorated by the feed additives, which may be related to the repair of the intestinal physical barrier.

To further explore the reparative effects of the five selected additives on SBMIE, we assessed the mRNA expression levels of the intestinal barrier and immune-related genes. Intestinal tight junction proteins and cytoskeletal factors are crucial for protecting the integrity and function of the intestinal barrier, acting as epithelial barriers, and protecting the intestinal tract from viruses and harmful bacteria (41). Hence, intestinal tight junction injury increases the chance of intestinal inflammation. Multiprotein tight junction complexes include occludin, claudin, and ZOs (42–44). In the present study, the additives remarkably increased the mRNA expression levels of tight junction factors (*ocln, zo1*) in the intestine of yellow drum, while reducing that of *mlck*. Notably, *mlck* phosphorylates MLC and regulates tight junction permeability (45, 46). Hence, *mlck* downregulation likely reduced intestinal permeability and improved the symptoms of SBMIE. The cytoskeleton is central to maintaining cellular function and structure, and regulating adherens junctions (AJ) (47, 48). In this study, the mRNA expression of cytoskeletal factors (*f-actin* and *arp2/3*) increased in the intestines of yellow drums fed additive-supplemented diets. The upregulation of intestinal tight junctions and cytoskeletal factors suggests that intestinal barrier injury is repaired, thereby reducing the risk of intestinal inflammation (49). This is consistent with findings in other marine fish (50).

Inflammatory and anti-inflammatory cytokines secreted by the intestinal immune effector cells mediate the intestinal inflammatory response. Although proinflammatory cytokines boost the differentiation of T and B cells, leading to inflammation (51), anti-inflammatory cytokines may also reduce the probability of intestinal inflammation (52). The pro-inflammatory factors Il-1 and Tnf-α act as upstream signals for other pro-inflammatory factors, promoting their production. In contrast, Il-10 and Tgf-β are key factors in preventing enteritis development (53). The results of the current study indicated that the expression of *il1*, *il6*, and *tnfa* mRNA was remarkably downregulated, and *il10* and *tgfb* were remarkably upregulated in fish fed the additive-supplemented diets compared to those fed the SBM diet. This may be due to the additives regulating immune response signaling pathways (Liu et al., 2018), which is in line with the findings of Giri et al. (2019) and Cheng et al. (2011) (26, 54, 55). However, fish fed the SBM-BBR and SBM-TPS diets did not exhibit significant changes in anti-inflammatory or pro-inflammatory cytokine levels, potentially due to the limited repair effect of berberine and tea polyphenols on yellow drum SBMIE, which resulted in incomplete intestinal injury repair.

In summary, the additives selected for this study elicited reparative effects on SBMIE symptoms in yellow drum. The SBMC diet (supplemented with curcumin) had the most reparative effect on growth performance, intestinal morphology, and gene expression of yellow drum. It is speculated that the selected additives enhance the immune function of yellow drum by regulating anti-inflammatory and pro-inflammatory factors, and improve intestinal morphology and nutrient absorption by elevating the expression of intestinal barrier related genes in yellow drum, thereby enhancing the growth performance of yellow drum and increasing its tolerance to high-level soybean meal feed. However, the specific mechanism of the reparative effects of dietary additives in treating SBMIE in yellow drum should be studied further.

## Data availability statement

The original contributions presented in the study are included in the article/supplementary material. Further inquiries can be directed to the corresponding authors.

## Ethics statement

The animal studies were approved by Zhejiang Ocean University’s Committee on Ethics of Animal Experiments. The studies were conducted in accordance with the local legislation and institutional requirements. Written informed consent was obtained from the owners for the participation of their animals in this study.

## Author contributions

SM: Writing – original draft, Writing – review & editing. LW: Writing – original draft, Writing – review & editing. YZ: Writing – original draft. PT: Writing – original draft. RC: Writing – original draft. WH: Writing – original draft. DX: Writing – review & editing, Writing – original draft. HX: Writing – review & editing.
